# Targeting Nrf2 and NF-κB Signaling Pathways in Inflammatory Pain: The Role of Polyphenols from Thinned Apples

**DOI:** 10.3390/molecules28145376

**Published:** 2023-07-13

**Authors:** Livia Interdonato, Giulio Ferrario, Marika Cordaro, Ramona D’Amico, Rosalba Siracusa, Roberta Fusco, Daniela Impellizzeri, Salvatore Cuzzocrea, Giancarlo Aldini, Rosanna Di Paola

**Affiliations:** 1 Department of Chemical, Biological, Pharmaceutical and Environmental Sciences, University of Messina, 98168 Messina, Italy; interdonatol@unime.it (L.I.); rdamico@unime.it (R.D.); rsiracusa@unime.it (R.S.); rfusco@unime.it (R.F.); salvator@unime.it (S.C.); 2 Department of Pharmaceutical Sciences (DISFARM), Università degli Studi di Milano, Via Mangiagalli 25, 20133 Milan, Italy; giulio.ferrario1@unimi.it (G.F.); giancarlo.aldini@unimi.it (G.A.); 3 Department of Biomedical, Dental and Morphological and Functional Imaging, University of Messina, Via Consolare Valeria, 98125 Messina, Italy; cordarom@unime.it; 4 Department of Veterinary Sciences, University of Messina, 98168 Messina, Italy; dipaolar@unime.it

**Keywords:** thinned apple polyphenols, paw edema, inflammation, oxidative stress, pain

## Abstract

Diet can modulate the different stages of inflammation due to the presence of bioactive compounds such as polyphenols. Apples are a great source of phenolic compounds that show anti-inflammatory and antioxidant properties, and these might be used as a dietary supplement and/or functional element in the treatment of chronic inflammatory illnesses. The aim of our study was to evaluate the anti-inflammatory and antioxidant actions of thinned apple polyphenol (TAP) extracts in a model of paw edema. The experimental model was induced in rats via subplantar injections of 1% λ-Carrageenan (CAR) in the right hind leg, and TAP extract was administered via oral gavage 30 min before and 1 h after the CAR injection at doses of 5 mg/kg and 10 mg/kg, respectively. The inflammatory response is usually quantified by the increase in the size of the paw (edema), which is maximal about 5 h after the injection of CAR. CAR-induced inflammation generates the release of pro-inflammatory mediators and reactive oxygen species (ROS). Furthermore, the inflammatory state induces the pain that involves the peripheral nociceptors, but above all it acts centrally at the level of the spinal cord. Our results showed that the TAP extracts reduced paw histological changes, neutrophil infiltration, mast cell degranulation, and oxidative stress. Additionally, the oral administration of TAP extracts decreased thermal and mechanical hyperalgesia, along with a reduction in spinal microglia and the markers of nociception. In conclusion, we demonstrate that TAP extract is able to modulate inflammatory, oxidative, and painful processes, and is also useful in the treatment of the symptoms associated with paw edema.

## 1. Introduction

One of the most often used methods for the screening and evaluating of anti-inflammatory medications is based on the capacity of such compounds to prevent the edema induced in the hind paw of a rat via the injection of a phlogistic agent. Inflammation is the first physiological response to tissue injury [1]. Acute inflammatory events cause physiological changes that serve to manage infection and return tissue to its healthy state. The acute inflammatory state is typically divided into four distinct sub-events: fluid exudation, which aids in the delivery of plasma proteins to sites of damage; neutrophil infiltration, which results in the removal of pathogens and cellular fragments; vasodilation, which aids in the delivery of necessary proteins and cells (similar to exudation), as well as increases tissue temperature; and pain and loss of function, which encourage rest and reduce the risk of further tissue damage [2]. CAR-induced paw edema is a well-known acute model of inflammation used to screen for novel anti-inflammatory drugs. CAR was injected into the subplantar surface of a rat paw, causing a biphasic edema. The early phase (around 1 h) was associated with the release of histamine, serotonin, bradykinin, and—to a lesser extent—the prostaglandins produced by cyclooxygenase enzymes (COX), whereas the delayed phase (after 1 h) was associated with neutrophil infiltration and the continuation of prostaglandin production [2,3]. The delayed phase of CAR-induced acute inflammation was likewise characterized by the release of neutrophil-derived free radicals, nitric oxide (NO), and pro-inflammatory cytokines [4,5]. The instability of the free radicals was primarily the result of an electron loss, which leads to increased reactivity and a persistent “theft” of electrons from other molecules, resulting in a deadly chain reaction known as “free radical damage” [6]. Since free radicals are significant mediators of inflammatory processes, their neutralization by antioxidants and radical scavengers can reduce inflammation. To reduce free radical damage, an organism will employ numerous enzymes such as superoxide dismutase (SOD) and catalase (CAT), as well as cofactors such as glutathione (GSH) [7,8]. Mast cells (MCs) are myeloid progenitor cells that are widely distributed in mucosal and connective tissues to alleviate inflammation by releasing pro-inflammatory chemicals such as histamine, proteases, proteoglycans, chemokines, arachidonic acid, and growth factors [9,10]. As a result, the quantity of mast cells in inflamed tissues reflects the prognosis and anti-inflammatory therapy efficacy. Many scientific studies have shown that there is a cross-talking between the inflammatory state and oxidative stress; this situation, marked by increased superoxide anion generation, propagates the inflammatory response, culminating in initial nociceptive sensitization [11,12]. Of paw edema, pain is an ever-present feature due to the formation of edema and the presence of an inflammatory state. Pain is a key indicator of inflammation, and it can be triggered by either the direct activation of nociceptors or by the activity of inflammatory mediators [13,14]. Remarkably, reactive oxygen and nitrogen species improve nociceptive transmission not only at the site of initial damage, but also at the spinal cord level in response to increased nociceptive transmissions following a peripheral injury. Primary sensory neurons detect painful stimuli at the periphery, as well as initiate and propagate the nociceptive signaling to spinal cord sites, where glial cells are activated in order to produce several mediators that induce nociceptor sensitization and neuroinflammation [15,16,17]. Spinal glial cells produce inflammatory cytokines and free radicals, particularly superoxide anion, in response to enhanced neurotransmission [18,19]. During the early stages of CAR-induced inflammation, both spinal microglial activity and microglial interleukin-1β (IL-1β) expression are elevated [20,21]. IL-1β is an early pro-inflammatory cytokine that can be generated by the activated microglia that can trigger inflammatory cascades or can modify the function of neighboring cells such as neurons and astrocytes, thus resulting in altered nociceptive processing. For this reason, limiting the production of inflammatory mediators is thought to be an effective therapeutic strategy for ameliorating inflammation pain [22,23].

Diet can modulate the different stages of inflammation due to the presence of bioactive compounds such as polyphenols, which are abundantly found in various food groups such as vegetables, fruits, nuts, cereals, and beverages. This makes them a class of secondary metabolites, which is an invaluable component in nutraceutical, pharmaceutical, and medicinal applications [24]. According to pre-clinical and intervention research, the apple is the fourth most important fruit that is farmed and consumed worldwide, and it is a rich source of bioactive anti-inflammatory polyphenols. Apple polyphenols exert antioxidant and anti-inflammatory activities, as demonstrated in different animal models, including non-alcoholic hepatitis [25], ulcerative colitis [26], and indomethacin-induced gastric damage [27]. The anti-inflammatory activity has also been found in humans: a regular consumption of 2–3 apples per day was associated with mitigating the inflammation in overweight and obese subjects by reducing the circulating biomarkers of inflammation and endotoxin exposure, including CRP, IL-6, and LBP, as well as by increasing the plasma antioxidant capacity [28]. At the molecular level, the antioxidant and anti-inflammatory effects of apple polyphenols can be explained in part by the polyphenols’ activity as Nrf-2 pathway activators [29]. As a result, the phenolic-enhanced fraction from apples provides a significant source of natural chemicals with anti-inflammatory and anti-oxidative stress properties that might be used as a dietary supplement and/or functional element in the treatment of chronic inflammatory illnesses. Currently, in view of a sustainable approach, there is a need to obtain bioactive dietary compounds that do not require food as a source but rather waste products that are derived from agriculture and from the food industry (circular economy) [30]. Consequently, there is a great interest in the waste products that derive from the apple waste chain. Among these, thinned young apples represent a massive waste product as they are usually discarded in orchard soil. Thinning apples, which is carried out around one month after blossom, is conducted in order to guarantee the output and to increase the quality of the harvested apples [31]. Thinned young apples are particularly rich in polyphenols, more than 10-fold with respect to harvested apples, and hence represent a suitable waste source for polyphenols [32].

We have recently reported in in vitro studies that thinned apple polyphenol (TAP) fractions (24% of polyphenols), which were obtained by purification through absorbent resins, show in cell models (with gene reporters for NRF2 and NF-κB) dose-dependent antioxidant and anti-inflammatory activities [33]. The effects were then further confirmed by proteomic studies, which elucidated the molecular pathways evoked by TAP treatment: the activation of the NRF2 signaling pathway. This, in turn, up-regulates the protective oxidoreductases and their nucleophilic substrates such as GSH and NADPH; the latter of which results from the up-regulation of the pentose phosphate pathway. The increase in the enzymatic antioxidant cellular activity together with the up-regulation of the heme-oxygenase explained the anti-inflammatory effect of TAP.

Based on these in vitro promising studies, the objectives of this study were to determine the anti-inflammatory and anti-nociceptive effects of thinned apple polyphenol (TAP) extract on a murine model of paw edema, as well as to explore its possible molecular mechanisms by the use of histological and molecular analyses.

## 2. Results

### 2.1. Effect of TAP Extract on CAR-Induced Inflammation and Pain

**Figure 1:**
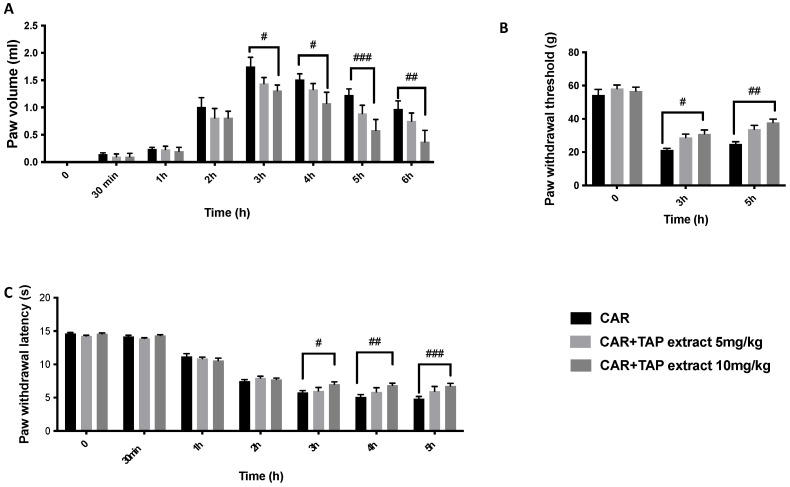
Evaluation of the effects of TAP extract on CAR-induced inflammation and pain. Paw volume (**A**); Von Frey test (**B**); and plantar test (**C**). Data are expressed as the means ± SEM of six animals from each group. # *p* < 0.05 vs. CAR; ## *p* < 0.01 vs. CAR; and ### *p* < 0.001 vs. CAR.

One of the initial signs of intraplantar CAR injection is a time-dependent increase in paw volume (Figure 1A), and this was measured at different timepoints between 0 (the start of the experiment) and 6 h (when the experiment ended). In our research, we discovered that the oral administration of TAP extracts at a dosage of 5 mg/kg was not able to reduce, in a significant way, the volume of paw edema. Meanwhile, 10 mg/kg resulted in a significant reduction in the volume of the rat paw, particularly at 6 h after CAR. In addition, CAR injection caused increased thermal hyperalgesia (Figure 1C) and mechanical allodynia (Figure 1B). The treatment with TAP extract at 10 mg/kg also significantly reduced thermal and mechanical hyperalgesia (Figure 1B,C).

### 2.2. Effects of TAP Extract on Histological Alteration after CAR Injection

**Figure 2:**
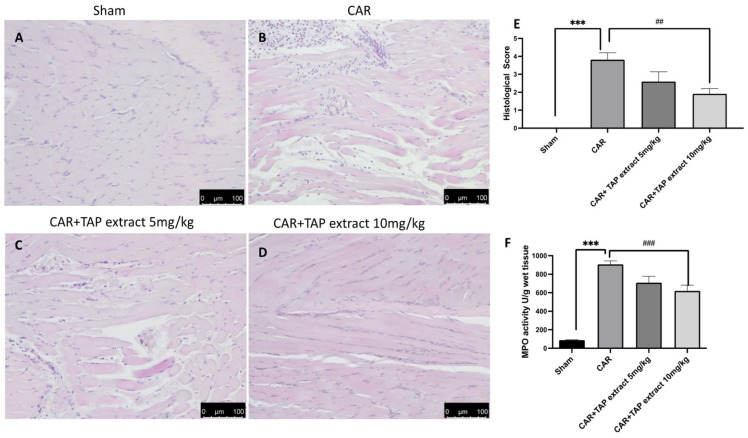
Histological evaluation of paw tissue. H/E staining was used to examine the tissue damage inflicted on the animals receiving CAR. Sham group (**A**), CAR group (**B**), and TAP extracts at the doses of 5 mg/kg and 10 mg/kg (**C**,**D**). Histological score (**E**). MPO analysis (**F**). Figures are representative of at least three independent experiments. Values are the means ± SEM of six animals from each group. Scale bar: 100 μm. *** *p* < 0.001 vs. sham; ## *p* < 0.01 vs. CAR; and ### *p* <0.001 vs. CAR.

A histological analysis of the paw tissue was conducted after the experiment by H/E inspection. The paw samples from the CAR group demonstrated edema formation, cellular diffuse infiltration, and a significant modification in the tissue architecture when analyzed microscopically (Figure 2). The paw tissues from the rat group that were treated with TAP extract treatment at a dose of 5 mg/kg (Figure 2C,D) still showed tissue alterations. On the contrary, a TAP extract at a dose of 10 mg/kg counteracted both cellular infiltration and edema production. The sham rats displayed a typical paw tissue architecture (Figure 2A). As a consequence of neutrophils infiltration, MPO was assessed. CAR induces a significant increase in MPO activity. On the other hand, oral treatment with a TAP extract at the dose of 10 mg/kg and not at 5 mg/kg significantly reduced MPO activity (Figure 2F).

### 2.3. Effect of TAP Extract on Mast Cell Activation after CAR Injection

**Figure 3:**
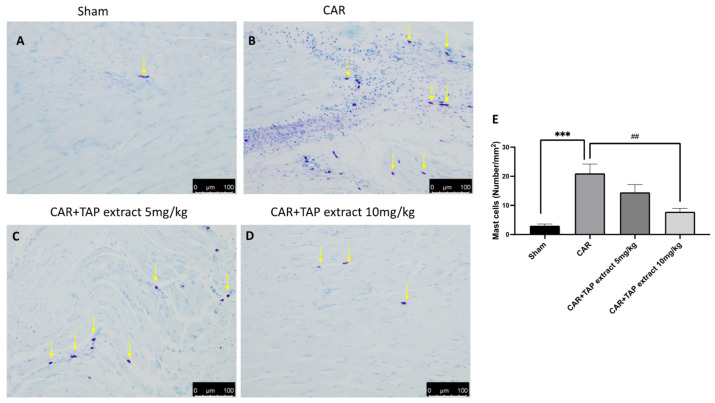
Histological evaluation of mast cell activity in paw tissue. Toluidine blue staining was used to examine the mast cell (yellow arrows) activation on the animals receiving CAR. Sham group (**A**), CAR group (**B**), and TAP extracts at the doses of 5 mg/kg (**C**) and 10 mg/kg (**D**). Mast cell count (**E**). Figures are representative of at least three independent experiments. Values are the means ± SEM of six animals from each group. Scale bar: 100 μm. *** *p* < 0.001 vs. sham; ## *p* < 0.01 vs. CAR.

Via toluidine blue staining, we detected increased mast cell recruitment at the lesion site when compared with the Sham group (Figure 3A). The administration of TAP extract at a dosage of 5 mg/kg (Figure 3C) reduced, but not significantly, the number of mast cells when compared to the CAR group (Figure 3B); meanwhile, the dose at 10 mg/kg was able to significantly reduce the number of mast cells present at the site (Figure 3D).

### 2.4. Effect of TAP Extract on Chymase and Tryptase Expression

**Figure 4:**
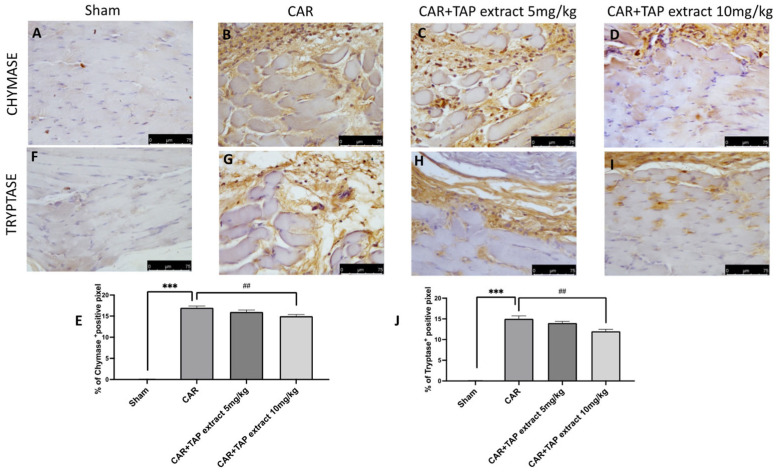
Immunohistochemical analysis in paw tissue. Immunohistochemical analysis was used to examine the mast cell activation that assesses the expression of tryptase and chymase, which are the markers of mast cell activation and degranulation on the animals receiving CAR. Sham group (**A**,**F**), CAR group (**B**,**G**), and TAP extracts at the doses of 5 mg/kg and 10 mg/kg (**C**,**D**,**H**,**I**). Percentages of the positive pixels of chymase and tryptase (**E**,**J**). Figures are representative of at least three independent experiments. Values are the means ± SEM of six animals for each group. Scale bar: 75 μm. ## *p* < 0.01 vs. CAR; and *** *p* < 0.001 vs. sham.

Via immunohistochemical analysis, we assessed the expression of chymase and tryptase, which are the markers of mast cell activation and degranulation. CAR injection significantly increased the positive staining for tryptase and chymase (Figure 4B,G) when compared with the Sham group (Figure 4A,F). The oral administrations of TAP extract at 10 mg/kg significantly decreased the expression of chymase and tryptase (Figure 4D,I).

### 2.5. Effect of TAP Extract Oral Administration on NF-κB, Nrf-2, and HO-1 Expression in Paw and Spinal Cord Tissues

**Figure 5:**
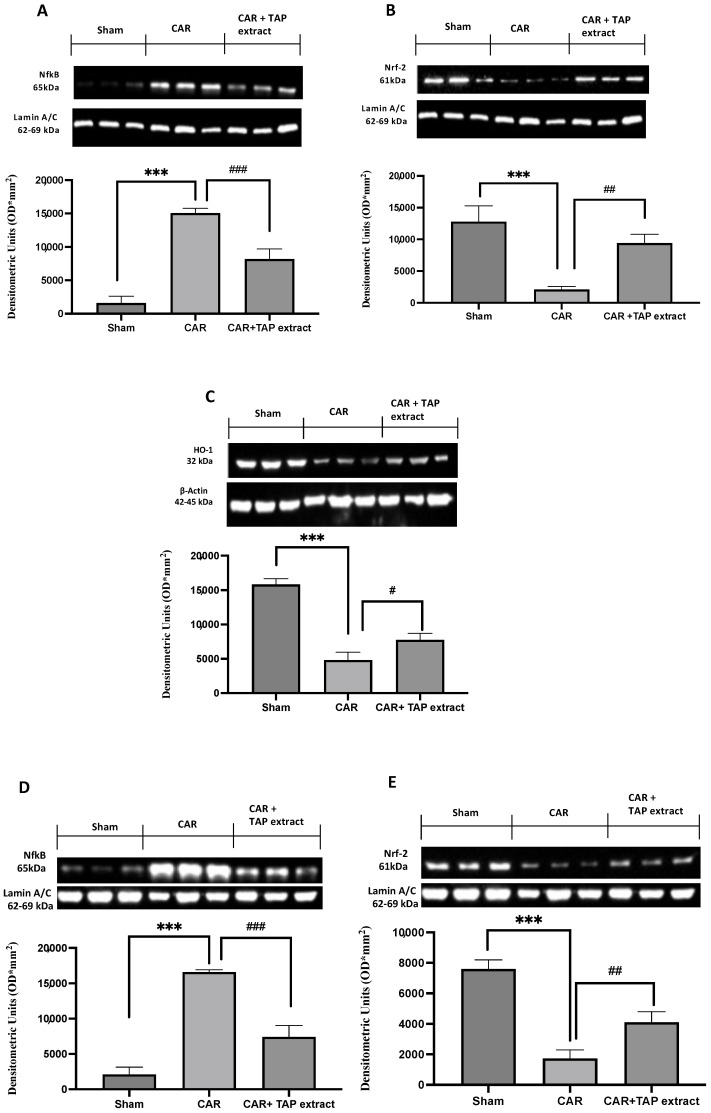

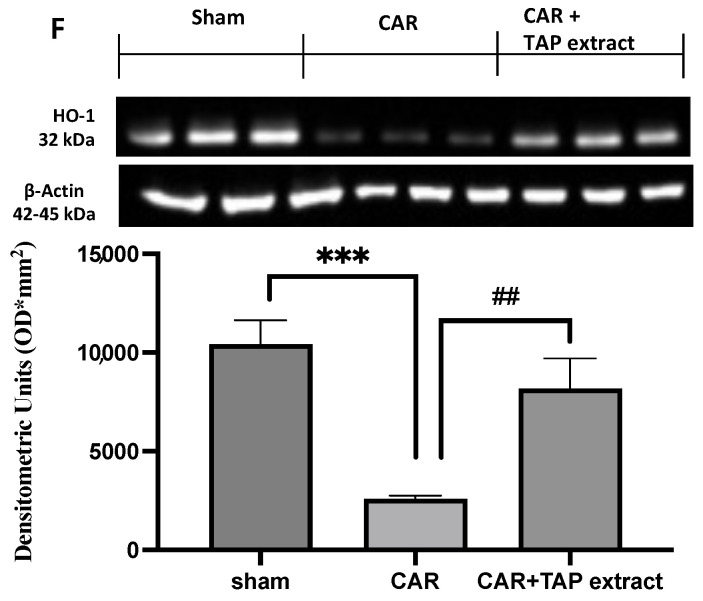
Western blot analysis in paw tissues and spinal cords. Western blot analysis for NF-κB paw expression (**A**), NF-κB spinal cord expression (**D**), Nrf-2 paw expression (**B**), NRF-2 spinal cord expression (**E**), HO-1 paw expression (**C**), HO-1 spinal cord expression (**F**), and TAP extract at a dose of 10 mg/kg. Values are the means ± SEM of six animals from each group. A demonstrative blot of lysates with a densitometric analysis for all animals is shown. # *p* < 0.05 vs. CAR; ## *p* < 0.01 vs. CAR; and ### *p* <0.001 vs. CAR. *** *p* < 0.001 vs. sham.

To better investigate whether TAP extract may act by interacting with signaling pathways such as nuclear NF-κB or Nrf-2/HO-1, Western blots for the NF-κB and NRF-2/HO-1 (Figure 5A–C expression in paw, Figure 5D–F expression in spinal cord) pathways were also performed in paw and spinal cord tissues. Increased nuclear NF-κB and reduced Nrf-2 expression were observed with respect to the sham animals. TAP extract significantly reduced the level of nuclear NF-κB, as well as up-regulated Nrf-2 when compared with the CAR group in both tissues. At the same time, Western blot analysis showed that TAP extract treatment at the dose of 10 mg/kg significantly enhanced a decrease in HO-1 protein expression in both tissues.

### 2.6. Effect of TAP Extract Oral Administration on IL-1β, Iba-1, and c-Fos Expression in Spinal Cord Tissues

**Figure 6:**
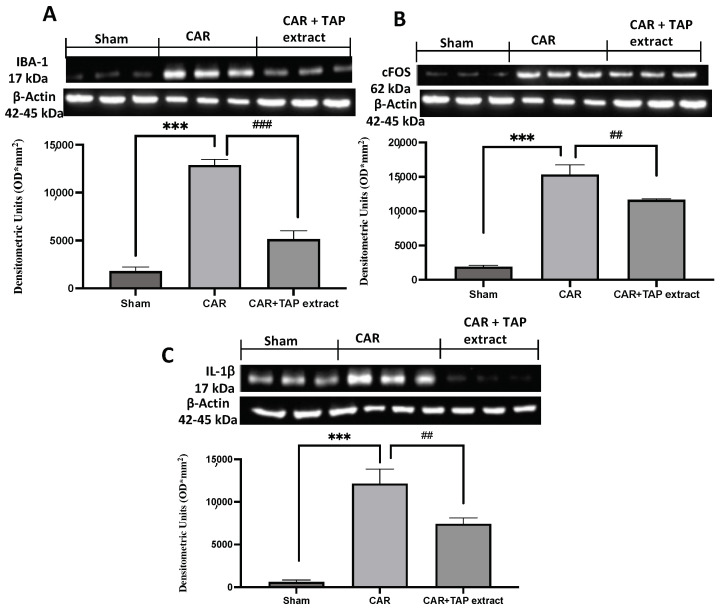
Western blot analysis in spinal cord. Western blot analysis for Iba-1 (**A**), c-Fos (**B**), and IL-1β (**C**). Values are the means ± SEM of six animals from each group. A demonstrative blot of lysates with a densitometric analysis for all animals is shown ## *p* < 0.01 vs. CAR; and ### *p* < 0.001 vs. CAR; *** *p* < 0.001 vs. sham.

As indicated above, spinal-activated microglia can generate IL-1β production, which is a pro-inflammatory cytokine that can trigger inflammatory cascades and modify the function of neighboring cells such as neurons, thus resulting in altered nociceptive processing. To examine the involvement of spinal microglia in this CAR model, the expression of Iba-1 (Figure 6A) in the spinal cord was quantified by Western blot. Compared with the expression level of Iba-1 in the sham animals, the expression level of Iba-1 in the CAR-injected rats was found to be significantly increased. Spinal Iba-1 and the expression of IL-1β (Figure 6C) were both up-regulated during CAR-induced inflammation. Due to the creation of edema and the existence of the inflammatory state, pain is an ever-present aspect; for this reason, we also measured changes in spinal c-Fos expression as a marker of neuronal activity (Figure 6B). The oral treatment of TAP extract at a dose of 10 mg/kg was able to modulate the expression of Iba-1 and Il-1β, as well as suppressed spinal c-Fos expression.

## 3. Discussion

An acute inflammatory reaction is defined as redness, heat, swelling, discomfort, and loss of function [7]. CAR-induced inflammation results in an acute and local inflammatory response, which is useful for orally detecting anti-inflammatory agents. The quest for natural compounds with antioxidant and anti-inflammatory properties has accelerated in recent decades owing to the fact that natural products are safe, efficacious, biocompatible, and cost-effective treatments for inflammatory illnesses [34]. Fruits and vegetables include a number of physiologically active metabolites that can be utilized in place of pharmaceuticals [35]. Polyphenolic chemicals are responsible for the color, flavor, and taste of plant-based meals, as well as for supposed health advantages for humans. Polyphenol concentrations are regulated by plant variety, as well as environmental conditions such as geographic area, growth season, and storage [36]. Apples are a great example of a fruit that has caught scholars’ curiosity for a number of reasons. Apples have a high nutritional content and a diverse range of bioactive components, making them a popular fruit in addition to being easily available, versatile, and cost-effective. Although apple consumption has been related to a number of positive health outcomes, few studies have looked into whether apples consumed in other forms, such as apple juice, pomace, cider, vinegar, and others, have the same health benefits as whole fruits [37]. Apple polyphenols are a promising bioactive material, and—in view of a sustainable approach—we have recently identified thinned apples as a waste source for obtaining an enriched apple polyphenol fraction (i.e., the TAP extract). We then fully characterized the qualitative composition of the TAP extract and demonstrated its anti-inflammatory and anti-oxidant activity in in vitro models [25]. Based on these studies, we evaluated the beneficial effect of TAP extract in a model of CAR-induced paw edema, and we investigated the molecular mechanisms involved in peripheral (paws) and central (spinal cord) tissues. The first step of acute inflammatory response is characterized by edema, which is often formed because of the exudation of fluid and plasma proteins [4,38,39]. Moreover, CAR-induced paw edema causes primary sensory neuron sensitization, which results in inflammatory discomfort. In humans, nociceptor sensitization often results in hyperalgesia, which is defined as an enhanced reaction to a painful stimulus, or allodynia, which is defined as pain triggered by non-noxious stimuli [40,41]. In this study, we demonstrated that taking TAP extract reduced hyperalgesia and allodynia 5 h later. After a CAR-induced acute inflammation event, paw tissue loses its normal muscle architecture, exhibits a significant accumulation of infiltrating inflammatory cells, and has increased inter-fiber space (when observed under microscopic examination) [42]. During our work, we discovered that the oral treatment of TAP extract at the dose of 10 mg/kg lowered the number of infiltrating inflammatory cells, which was also evidenced by the considerable drop of MPO in our analysis [43]. MC activity modulates vascular permeability and the accumulation of cells during inflammatory response, as well as stimulates afferent chemosensitive fibers in the skin to release neuropeptides that promote neurogenic inflammation [44,45]. In this regard, the anti-inflammatory properties of orally administrated TAP extract at a dose of 10 mg/kg was mainly due to the control of MC activation. Moreover, as expected, our results confirmed a reduced number of MCs after the treatment [46]. Consequently, we observed an important reduction in chymase and tryptase expression after TAP extract oral administration, confirming the control on MC activity. NF-κB is the main intracellular pathway involved in the inflammatory response [47]. NF-κB is a protein complex that acts as a transcription factor, which covers a key role in inflammatory processes and many other diseases [48]. The IKK complex, which consists of three subunits, catalyzes the phosphorylation of IkBα. Among them, IKKβ is the catalytic component that phosphorylates IκB for destruction via ubiquitination [49]. The nuclear translocation of NF-κB increases the transcription of the particular genes involved in the generation of pro-inflammatory cytokines [50,51]. To see if TAP extract affects NF-κB activity, we used Western blotting to look at the expression of the NF-κB pathway in paw tissues. Our findings revealed that p65NF-κB levels increased significantly after CAR therapy, and these were lowered with TAP extract pretreatment. Neutrophils generate a significant amount of superoxide anion, which contributes to many of the negative consequences of inflammation, such as oxidative stress, tissue damage, and hyperalgesia. One of the most significant consequences of high oxidative stress is cell damage caused by ROS [52]. In response to various types of stimulation, such as oxidative stress, Nrf2 is the major transcriptional activator of the HO-1 gene [53]. Nrf2 is an important mediator of the body’s endogenous inducible defensive mechanisms. Under physiological and environmental circumstances, Nrf2 is anchored in the cytoplasm by Kelch-like ECH-associated protein 1 (Keap1) and is destroyed via the ubiquitin proteasome pathway [54]. In response to oxidative stress, Nrf2 is produced by Keap1 and translocated to the nucleus, where it binds to antioxidant response elements (ARE) in order to induce the transcription of cytoprotective genes such as hemeoxygenase-1 (HO-1) [23,55]. Western blot analysis revealed that the Nrf2 expression was lower in the paw tissues of the CAR group when compared to the sham group. TAP extract treatment was able to enhance Nrf2 nuclear translocation, as well as induced the expression of Nrf2 and regulatory factors such as HO-1. Pain sensitivity increases as a result of tissue damage, which is a frequent component of the inflammatory response. NF-κB-related pro-inflammatory mediators, such as IL-1β, cause peripheral sensitization [56]. To highlight the link between the CNS and the periphery, we also performed Western blot analysis of the NF-κB and Nrf2 in spinal cords. Our results showed that these two pathways were altered after CAR injection and were reestablished by TAP extract administration at a dose of 10 mg/kg. Spinal microglia are activated early after noxious insults, and they can produce a number of mediators that modify the CNS microenvironment. Microglial cells contribute to hyperalgesia by producing nociceptive molecules such as cytokines like IL-1β, which increases nociceptive transmission [15]. Spinal IL-1β is an important factor in the regulation of nociceptive processing in the CNS [57]. IL-1β and Iba-1 are markers of microglia activation, and they are elevated during the early stages of peripheral inflammation, which can impact the activity of several neighboring cells in the dorsal horn of the spinal cord [57]. Accordingly, our data showed an increased expression of IL-1β and Iba-1 in the spinal cords of CAR-injected rats that were reduced by the administration of TAP extract. CAR injection into the plantar area of the rat hind paw stimulates c-Fos mRNA and Fos protein expression in the lumbar spinal cord [58]. Several analgesics reduce the Fos rise caused by CAR, demonstrating that Fos expression is a marker of pain pathway activity and may be used to quantify analgesia. In this regard, our results also demonstrated that TAP extract reduced c-Fos expression as a marker of nociception.

## 4. Materials and Methods

### 4.1. Animals

All experiments were conducted using male Sprague Dawley rats (200–230 g, Envigo, Milan, Italy). The study was given the go-ahead by the University of Messina’s OPBA (Animal Care Review Board). All animal experiments adhere to EU Directive 2010/63, and the new Italian legislation (D. Lgs. 2014/26).

### 4.2. CAR-Induced Paw Edema

Rats were given a subplantar injection of CAR (0.1 mL/rat of a 1% suspension in saline) with a 27-gauge needle into the right hind paw following anesthesia with 5.0% isoflurane in 100% O_2_ (conducted in the same manner as previously described by Morris and Britti [59,60]). The animals were killed by isoflurane overdose six hours after the CAR injection. All analyses were carried out using experimental groups in a blinded fashion [61]. Paw and lumbar spinal cord tissues were collected.

### 4.3. Experimental Groups

Rats were divided into the following groups at random (total number of animals was 18 rats for each experimental group, and 6 animal/group for diverse analyses):

1. CAR + vehicle (saline): the rats were subjected to CAR-induced paw edema;
2. CAR + TAP extract: the rats were subjected to CAR-induced paw edema and the TAP extract was orally administered 30 min before and 1 h after at doses of 5 mg/kg and 10 mg/kg;
3. Sham-operated: the rats underwent the same surgical procedures as the CAR group, with the exception that saline or drugs were administered instead of CAR;
4. CAR + indomethacin: the rats were subjected to CAR-induced paw edema and indomethacin was orally administered 30 min before and 1 h after at doses of 10 mg/kg; (see Figures S1 and S2 in Supplementary Materials).

The tested dose was selected based on earlier experiments carried out in our laboratory. See the preliminary results on the effect–dose response of TAP extract in Supplementary Figures S1 and S2 (see Supplementary Materials).

### 4.4. Assessment of CAR-Induced Paw Edema

Edema was evaluated in the manner previously mentioned in [59]. The volume of the paw was measured using a plethysmometer (Ugo Basile, Comerio, Italy) prior to the injection of CAR and for the following 6 h at hourly intervals. Edema was measured for each animal as an increase in paw volume (mL) following a CAR injection, and this was compared to the pre-injection value.

### 4.5. Pain-Related Behavioral Analysis in CAR-Induced Inflammation

The electronic von Frey test (Bio-EVF4; Bioseb, Vitrolles, France) was used to assess mechanical allodynia. The gadget has a controllable force transducer with a plastic tip. When pressure is applied to the tip, the electronic gadget automatically records the maximum force exerted (in grams) and displays it on the screen. The tip was placed on the plantar part of the hind leg, and an upward push was delivered until the paw was removed. The withdrawal threshold was defined as the force, measured in grams, with which the mouse retracted its paw. The withdrawal was calculated three times, and the stated figure is the average of the three calculations [62].

### 4.6. Histological Examination of the CAR-Inflamed Hind Paw

To evaluate mast cell (MC) degranulation, hematoxylin/eosin (H/E) staining and toluidine blue staining were performed and viewed blind in the treatment regimen. At the end of the experiment, paw tissues were dried, embedded in Paraplast, cut into 7 m slices, and examined under microscopy (Leica DM7, Milan, Italy). The severity of inflammation was measured using a 6-point scale: none, mild, mild/moderate, moderate, moderate/severe, and severe inflammation [63,64].

### 4.7. Myeloperoxidase (MPO) Activity

The paw tissues were homogenized in a 0.5 percent hexadecyltrimethylammonium bromide mixed in a 10 mM potassium phosphate buffer (pH 7.0), and were centrifuged at 20,000× *g* for 30 min at 4 °C. A supernatant aliquot was allowed to react with a solution of 1.6 mM of tetramethylbenzidine/0.1 mM of H_2_O_2_. A spectrophotometer was used to measure the rate of absorbance change at 650 nm. MPO activity was defined as the quantity of enzymes that degraded 1 mM of peroxide in 1 min at 37 °C, and this was represented in units per gram of wet tissue weight [65,66].

### 4.8. Immunohistochemical Localization of Chymase and Tryptase

The immunohistochemical evaluation for chymase and tryptase was realized as previously described in [67,68]. The slices were incubated overnight with an anti-Chymase mouse monoclonal antibody (Santa Cruz Biotechnology, Heidelberg, Germany; 1:100 in PBS, *v*/*v*) and anti-Tryptase mouse monoclonal antibody (Santa Cruz Biotechnology; 1:100 in PBS, *v*/*v*). The samples were washed with PBS and incubated with secondary antibodies. Specific labeling was identified with a biotin-conjugated goat anti-rabbit IgG and avidin–biotin peroxidase complex (Vector Laboratories, Burlingame, CA, USA) [69]. The stained sections were observed using a Leica DM6 microscope (Leica Microsystems S.p.A., Milan, Italy), following a typical procedure [70,71].

### 4.9. Western Blots Analysis

Western blot examination of the paw and spinal cord tissues was prepared as previously described in [72]. The following primary antibodies were used (for the purpose of standardization): anti-NF-κB (1:500), anti-Nrf-2 (1:500), anti-HO-1 (1:500), anti-IL-1β (1:500), anti-c-Fos (1:500), anti-βactin (1:500), and β-laminin (1:500) (Santa Cruz Biotechnology, Heidelberg, Germany) [73]. Protein expression was quantified via densitometry with Bio-Rad ChemiDocTM XRS+ software (Bio-Rad, Milan, Italy) and normalized with the housekeeping genes β-actin and lamin A/C, as previously reported in [74,75].

### 4.10. Data Analysis

All values are the mean standard errors of the means ± (SEM) of N observations. The photos displayed are indicative of at least three tests that were performed on the tissue slices obtained from all animals in each group on different experimental days. N denotes the number of animals utilized in in vivo investigations. A one-way ANOVA was used to examine the data, followed by a Bonferroni post hoc test for multiple comparisons. A *p*-value of less than 0.05 was deemed significant. A *p* value of less than * *p* <0,05 vs. sham; # *p* < 0.05 vs. CAR; ** *p* < 0.01 vs. sham; ## *p* < 0.01 vs. CAR; *** *p* < 0.001 vs. sham; and ### *p* <0.001 vs. CAR.

### 4.11. Thinned Apple Polyphenol (TAP) Extract

The TAPs were isolated from thinned Golden, Fuji, Bella del Bosco, and Rosa Mantovana apples (sourced from the farms located in Trentino-Alto Adige, Italy) harvested 1 month after blossoming and stored at 2 °C for 1 month, as previously reported in [33]. Briefly, after washing with an aqueous solution containing 0.05% citric acid, the apples were coarsely ground in a hammer mill, and the resulting mush was added to a 0.1% solution of pectinase, which was then heated in a linear tunnel at 25 °C for 20 min. The mass was then continuously forced through a filter, and the juice was clarified by centrifugation. The resulting colorless solution was then eluted through an AMBERLITE XAD7 absorbent resin and washed with demineralized water until the elimination of all the substances that were not retained. The absorbed polyphenols were then eluted with 95% ethanol, and the hydro-alcoholic solution was concentrated under vacuum and then micronized.

The qualitative profile of the polyphenol components of TAP (24% of polyphenols) was evaluated by a targeted and untargeted metabolomic approach via HPLC-HRMS in the negative and positive ion mode, as already reported in [33]. A total of 68 compounds were identified: 52 by targeted and 19 by an untargeted approach. Of the 68 identified compounds, 23 were phenolic and organic acids, 11 were flavanols, 19 were flavonols, 6 were flavanones, 5 were dihydrochalcones, 1 was a flavone (luteolin), 1 was a triterpenoid (euscaphic acid), and two were lipids.

The quantitative content was determined both by spectrophotometry (Folin–Ciocalteu colorimetric test) and HPLC analysis [33]. Overall, the results derived by these two methods are superimposable, being 24.14 ± 1.58 and 27.97 ± 0.68 mg/100 mg, as determined by the Folin–Ciocalteu colorimetric test and HPLC analysis, respectively (see further details in Table S1 in Supplementary Materials).

## 5. Conclusions

**Figure 7:**
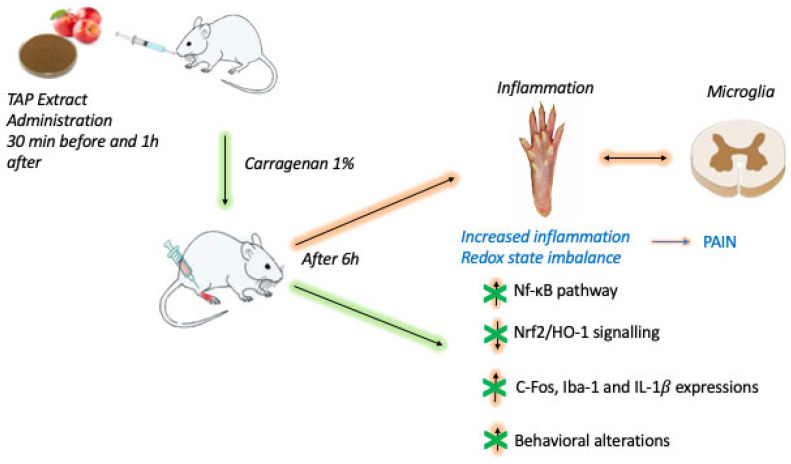
Schematic diagram on the evaluation of TAP extract oral administration in a CAR-induced paw edema experimental model.

Inflammation research has been a major focus of global scientific inquiry. Inflammation is recognized as being associated with oxidative processes, owing to the fact that they share certain similar pathways. As oxidative stress and inflammation are widespread in many degenerative diseases, dietary antioxidants may illuminate a significant protective impact. TAP extract is abundant in antioxidants, polyphenols, and other compounds that have been shown to be active. In conclusion, we demonstrated that orally administering TAP extract at a concentration of 10 mg/Kg was effective in considerably counteracting CAR-induced paw tissue damage and the resulting inflammatory pain (Figure 7).

## Data Availability

The data used to support the findings of this study are available from the corresponding author upon request.

## Supplementary Materials

The following supporting information can be downloaded at: https://www.mdpi.com/article/10.3390/molecules28145376/s1, Figure S1: Evaluation of the effects of TAP extract on CAR-induced inflammation and pain; Figure S2: MPO analysis; Figure S3: Preliminary results on the effect of dose response of TAP extract; Table S1: Qualitative profile of the TAP extract.

## Sample Availability

Samples of the compounds are available from the authors upon request.
