# Supplementary material for: Targeting Nrf2 and NF-κB Signaling Pathways in Inflammatory Pain: The Role of Polyphenols from Thinned Apples
Source: Molecules. 2023 Jul 13;28(14):5376. doi: 10.3390/molecules28145376 (PMC10385557; doi:10.3390/molecules28145376)
Supplement: Supplementary file 1 [file molecules-28-05376-s001.zip › molecules-2437770-supplementary.pdf]

Supplementary Materials

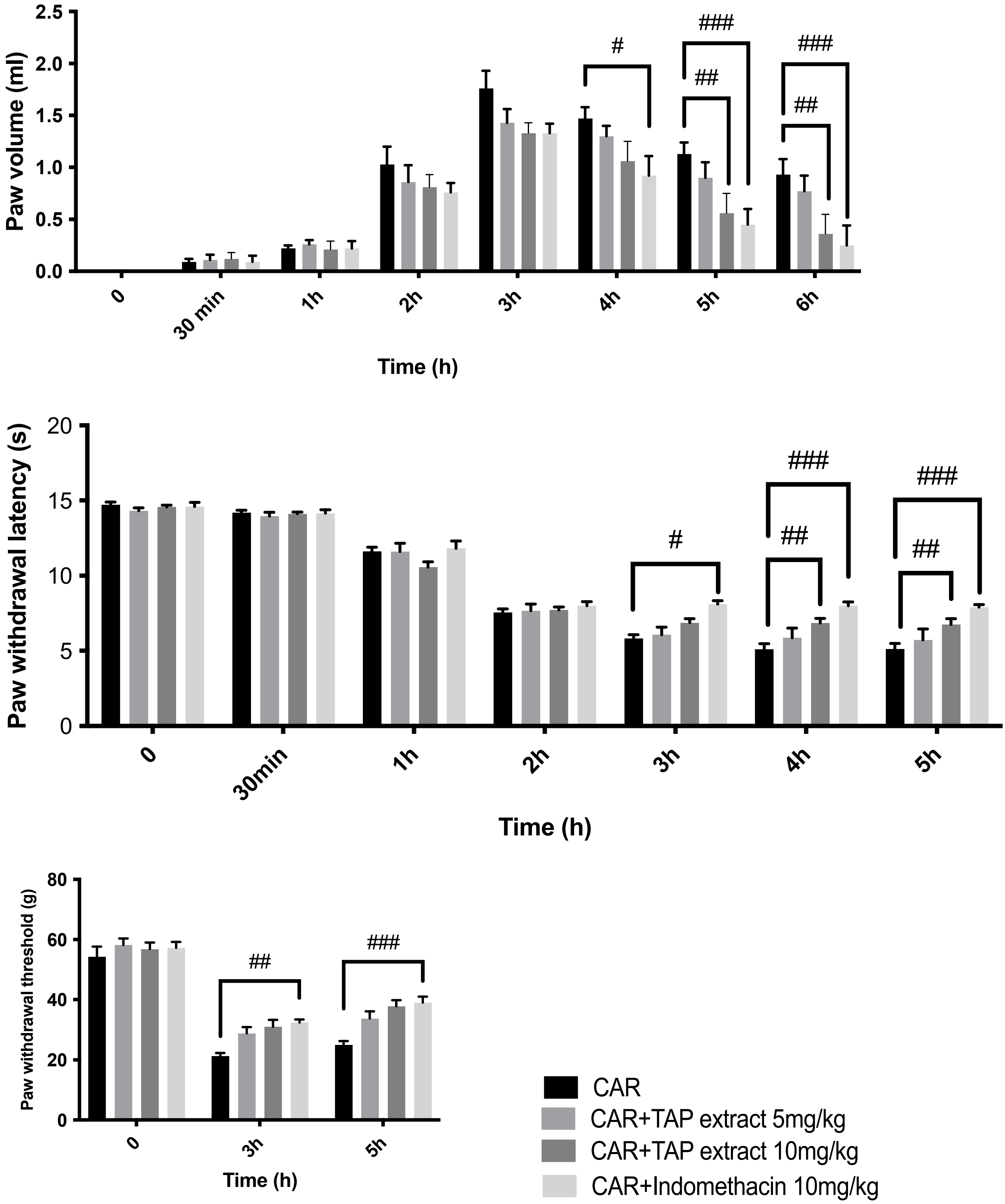

Figure S1. Evaluation of the effects of TAP extract on CAR-induced inflammation and pain. Paw volume; Von Frey test; plantar test. Data are expressed as means ± SEM of 6 animals for each group. # $p < 0.05$ vs. CAR; ## $p < 0.01$ vs. CAR; ### $p < 0.001$ vs. CAR.

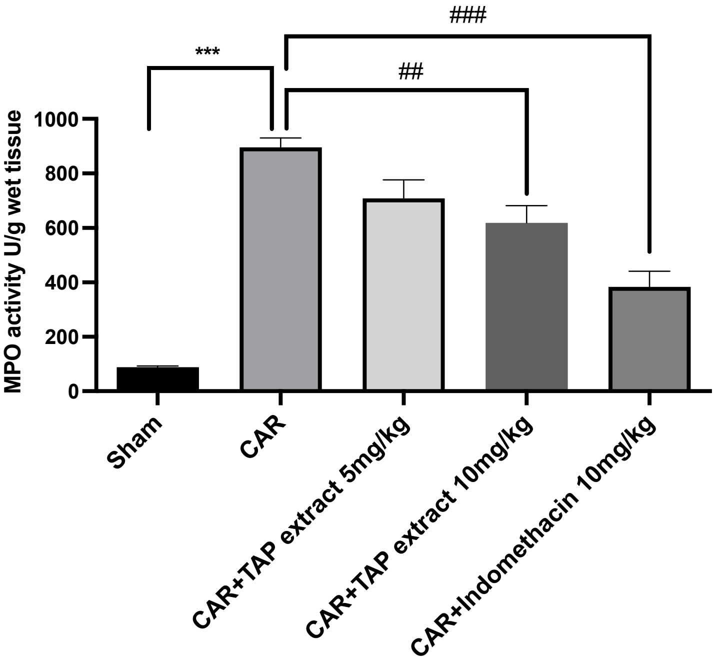

Figure S2. MPO analysis. Values are means ± SEM of 6 animals for each group. *** $p < 0.001$ vs. sham; ## $p < 0.01$ vs. CAR; ### $p < 0.001$ vs. CAR.

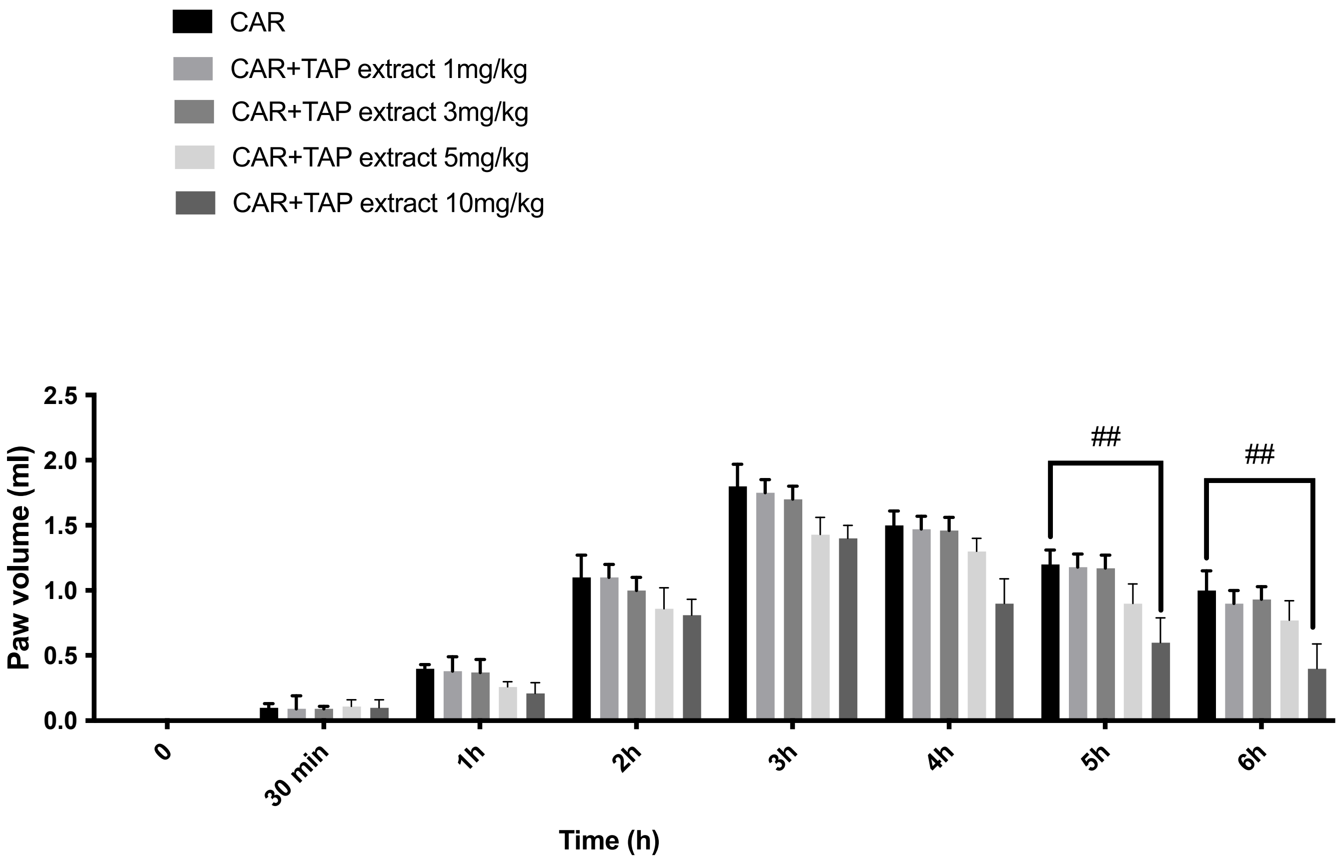

Figure S3: Preliminary results on the effect of dose response of TAP extract. Paw volume; Data are expressed as means ± SEM of 6 animals for each group. ## $p < 0.01$ vs. CAR;

Table 1. Qualitative profile of the TAP extract.

Organic and Phenolic Acids	
**Citric acid**	Caffeic acid
Protocatechuic acid glucoside	Ferulic acid-O-glucoside isomer 1
Caffeoylquinic acid isomer 1	*p*-Coumaric acid-O-glucoside isomer 3
Protocatechuic acid	Ferulic acid-O-glucoside isomer 2
Glutathionyl chlorogenic acid	Coumaroylquinic acid isomer 2
Caffeoyl glucoside isomer 1	Ferulic acid-O-glucoside isomer 3
Caffeoyl glucoside isomer 2	*p*-Coumaric Acid
Coumaroylquinic acid isomer 1	Dicaffeoylquinic acid
Caffeoyl glucoside isomer 3	3-(benzoyloxy)-2-hydroxypropyl glucopyranosiduronic acid
Caffeoylquinic acid isomer 2	Sinapoyl hexoside
p-Coumaric acid-O-glucoside isomer 1	3-[2,4,5-trihydroxy-3-(3-methylbut-2-en-1-yl) phenyl] propanoic acid
p-Coumaric acid-O-glucoside isomer 2	
Flavanols	
Catechin	Procyanidin pentamer
Procyanidin B2	Procyanidin hexamer
Epicatechin	Procyanidin heptamer
Procyanidin trimer	Procyanidin octamer
Procyanidin tetramer	Procyanidin nonamer
Procyanidin trimer	
Flavanones	
Naringenin glucoside (prunin)	Eriodictyol
Eriodictyol-hexoside	Naringenin
Naringin (Naringenin 7-O-neohesperidoside)	Eriodictyol 7-(6-trans-*p*-coumaroylglucoside)
Flavonols	
Kaempferol-glucoside	Patuletin hexoside isomer 1
Quercetin-3-O-glucoside	Patuletin hexoside isomer 2
Quercetin-3-O-galactoside	Patuletin hexoside isomer 3
Quercetin pentoside isomer 1	Patuletin pentoside
Quercetin pentoside isomer 2	Patuletin rhamnoside
Quercetin pentoside isomer 3	Isorhamnetin rhamnoside
Quercetin-3-O-rhamnoside	Kaempferol rhamnoside
Quercetin	Methoxyquercetin (patuletin)
Kaempferol	Quercetin 3-(3-*p*-coumaroylglucoside)
Isorhamnetin	
Dihydrochalchones	
Hydroxyphloretin glucoside	Phloridzin
Phloretin-O-xyloglucoside isomer 1	Phloretin
Phloretin-O-xyloglucoside isomer 2	
Flavones	
Luteolin	
Triterpenoids	
Euscaphic acid	
Lipids	
(10E,15Z)-9,12,13-Trihydroxy-10,15-octadecadienoic acid	Trihydroxy-octadecenoic acid
